# Reviewers Acknowledgment

**Published:** 2020

The Editor-in-Chief of *Asian/Pacific Island Nursing Journal* would like to thank the following people who acted as peer-reviewers for the journal from January 2019 through December 2019.

Patricia Alpert

Amnatsatsue Kwanjai

Balakrishnan Umamaheswar

Booranasuksakul Uraipron

Nafanua Braginsky

Byon Ha Do

Chen Wei-Ti

Chad Cross

Alona Dalusung-Angosta

Felicitas dela Cruz

Raymond Folen

Faye Gary

Artemio Gonzales

Margaret Hattori-Uchima

Margaret Heitkemper

Naoko Hikita

Dianne Ishida

Ira Kantrowitz-Gordon

Merle Kataoka-Yahiro

Karina Katigbak

Masashi Kawano

Jennifer Kawi

May Kealoha

Heeyoung Lee

Dongmei Lee

Wen-Wen Lee

Rebecca Lorenz

Nada Lukkahatai

May-Ying Ly

Jennifer Nevers

Angelina Nguyen

Na-Jin Park

Andrew Reyes

Karol Richardson

Reiko Sakashita

Todd Seto

Setyowati

Lillian Tom-Orme

Tsai Hsiu-Min

Nancy Woods

Joyce Yang

Yonezawa Kaori

